# Is HIV Infection a Risk Factor for Multi-Drug Resistant Tuberculosis? A Systematic Review

**DOI:** 10.1371/journal.pone.0005561

**Published:** 2009-05-15

**Authors:** Sujit Suchindran, Emily S. Brouwer, Annelies Van Rie

**Affiliations:** 1 School of Medicine, School of Public Health, University of North Carolina, Chapel Hill, North Carolina, United States of America; 2 Department of Epidemiology, School of Public Health, University of North Carolina, Chapel Hill, North Carolina, United States of America; University of Stellenbosch, South Africa

## Abstract

**Background:**

Tuberculosis (TB) is an important cause of human suffering and death. Human immunodeficiency virus (HIV), multi-drug resistant TB (MDR-TB), and extensive drug resistant tuberculosis (XDR-TB) have emerged as threats to TB control. The association between MDR-TB and HIV infection has not yet been fully investigated. We conducted a systematic review and meta-analysis to summarize the evidence on the association between HIV infection and MDR-TB.

**Methods and Results:**

Original studies providing *Mycobacterium tuberculosis* resistance data stratified by HIV status were identified using MEDLINE and ISI Web of Science. Crude MDR-TB prevalence ratios were calculated and analyzed by type of TB (primary or acquired), region and study period. Heterogeneity across studies was assessed, and pooled prevalence ratios were generated if appropriate. No clear association was found between MDR-TB and HIV infection across time and geographic locations. MDR-TB prevalence ratios in the 32 eligible studies, comparing MDR-TB prevalence by HIV status, ranged from 0.21 to 41.45. Assessment by geographical region or study period did not reveal noticeable patterns. The summary prevalence ratios for acquired and primary MDR-TB were 1.17 (95% CI 0.86, 1.6) and 2.72 (95% CI 2.03, 3.66), respectively. Studies eligible for review were few considering the size of the epidemics. Most studies were not adjusted for confounders and the heterogeneity across studies precluded the calculation of a meaningful overall summary measure.

**Conclusions:**

We could not demonstrate an overall association between MDR-TB and HIV or acquired MDR-TB and HIV, but our results suggest that HIV infection is associated with primary MDR-TB. Future well-designed studies and surveillance in all regions of the world are needed to better clarify the relationship between HIV infection and MDR-TB.

## Introduction

The Human Immunodeficiency Virus (HIV) pandemic is one of the greatest challenges facing tuberculosis (TB) control. Immune suppression increases the risk of reactivation of latent TB infection and rapid progression to active TB disease [1]. TB diagnosis is more difficult in people living with HIV infection and initiation of HIV treatment can paradoxically worsen TB by restoring immune function [2]. The effects of HIV on TB result in a strong correlation between HIV prevalence and TB incidence rates [3]. Overall, an estimated 8% of new TB cases are attributable to HIV co-infection [4]. The TB incidence rate and HIV prevalence among new TB cases is highest in sub-Saharan Africa. The leading cause of death among HIV infected patients in the developing world is TB. An estimated 13% of the 1.5 million TB deaths in 2006 were attributed to HIV infection, but in the African region this proportion has been much higher [3]. The risk of death in co-infected patients is twice that of HIV–infected individuals without TB, even when CD4+ cell count and antiretroviral therapy are taken into account [5].

Multidrug resistant tuberculosis (MDR-TB), defined as *Mycobacterium tuberculosis* resistant to isoniazid and rifampin, further threatens TB control because of high treatment failure and death rates, and complexities in diagnosis and treatment. MDR-TB can be a result of failure of drug sensitive TB treatment with development of resistance (acquired MDR-TB) or direct transmission of an MDR strain (primary MDR). Acquisition can arise from medical error, poor TB control programs or poor patient adherence to treatment. A history of prior TB treatment remains the most important risk factor for MDR-TB [6]. According to the most recent WHO estimates, 490,000 MDR-TB cases and more than 110,000 MDR-TB deaths occur annually [4]. The first international MDR-TB survey demonstrated that MDR-TB was present worldwide, with ‘hot-spots’ in Russia, Latvia and the Dominican Republic [7], [8]. The 2008 report, which included data until 2007, recorded the highest rates of MDR-TB ever, observed primary MDR-TB rates greater than 6% in 14 regions, and warned about extensively drug-resistant TB (XDR-TB, resistance to rifampin, isoniazid plus resistance to any fluoroquinolone and any of the second-line anti-TB injectable drugs) in 45 countries [9].

Given the dynamic interplay between HIV and TB, it is not surprising that MDR-TB has complicated the picture. HIV and MDR-TB are an even deadlier combination. More than 50% of HIV-infected MDR-TB patients in Peru died within two months of diagnosis [10] and studies with longer follow up observed death rates ranging from 72 to 89% [11]. A study in the UK estimated that MDR-TB patients who are immune-compromised are nine times more likely to die than those not immune-compromised [12]. In a XDR-TB outbreak in South Africa, 98% of co-infected patients died with median survival time of 16 days from XDR-TB diagnosis [13].

Even though the impact of HIV infection on MDR-TB is of great public health importance, their relationship is not yet fully understood. HIV infection has been associated with MDR-TB outbreaks in institutional settings, such as hospitals and prisons [14], [15]. It remains less clear whether HIV infection is also associated with MDR-TB in community settings. We aimed to summarize and critically appraise studies in order to quantify the association between MDR-TB and HIV-infection.

## Methods

**Figure 1:**
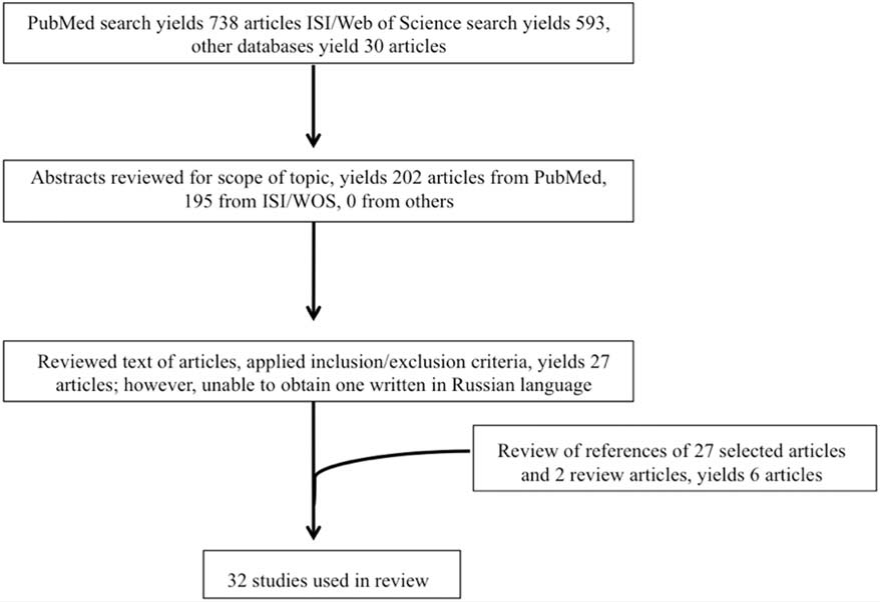
Search strategy.

A systematic review of studies assessing HIV infection as a risk factor for MDR-TB was carried out using MEDLINE/PubMed, ISI Web of Science, CINAHL and the Cochrane Library databases. The keywords “multi drug resistant tuberculosis”, “MDR-TB” and “HIV” were used as search terms. Searches were performed during March 2007 and included articles on human subject research prior to the search date. We reviewed titles and abstracts of original studies retrieved by the search, full text and references of selected articles, and text and references of relevant review articles published prior to the April 2007 (Figure 1).

Reports on original studies were included, independent of study design and without restriction of publication date, because of the low number of eligible studies (Table 1). Articles were included if they presented results of drug susceptibility to rifampin and isoniazid of *Mycobacterium tuberculosis*, stratified by HIV test result, and when values pertaining to the prevalence odds or prevalence rate for MDR-TB were extractable from data presented. Studies on children were not specifically excluded but none of the eligible published articles presented child data.

**Table 1:** MDR-TB prevalence by HIV status in 32 studies.

Ref.	Country	MDR-TB type	Study period	Number of HIV+ patients	MDR-TB in HIV+ patients	Number of HIV− patients	MDR-TB in HIV− patients
[20]	Thailand	Any	2000	192	5.2%	685	0.4%
[21]	Thailand	Primary	2001	377	8.5%	474	4.4%
		Acquired		49	40.8%	85	32.9%
[22]	India	Primary	00–'04	30	10.0%	40	2.5%
[23]	Vietnam	Primary	98–'00	40	7.5%	1393	3.7%
		Acquired		11	9.1%	390	25.9%
[24]	Cote d'Ivoire	Any	1989	17	0.0%	29	3.4%
[25]	Tanzania	Primary	91–'93	275	0.4%	816	0.4%
		Acquired		21	0.0%	52	3.8%
[26]	South Africa	Any	91–'94	42	2.4%	253	11.5%
[27]	South Africa	Primary	94–'96	93	3.2%	115	2.6%
[28]	South Africa	Any	1995	207	5.3%	215	6.5%
[29]	Botswana	Any	95–'96	117	0.9%	123	0.8%
[30]	Mozambique	Any	98–'99	179	2.2%	530	3.2%
[31]	Italy	Any	90–'92	34	2.9%	373	5.9%
[32]	Portugal	Any	95–'98	29	44.8%	113	17.7%
[33]	Spain	Primary	88–'92	184	0.5%	317	0.6%
[34]	Spain	Any	'96–'00	59	0.0%	926	0.1%
[35]	France	Primary	'92–'94	893	1.2%	5864	0.3%
		Acquired		107	11.2%	868	6.6%
[36]	France	Primary	95–'97	246	0.0%	2007*	0.4%
		Acquired		28	7.1%	226*	3.5%
[37]	United Kingdom	Any	93–'99	910	4.6%	24307*	1.1%
[38]	United Kingdom	Primary	93–'94, '98–'00	274	3.6%	7936*	1.0%
		Acquired		19	21.1%	611*	8.2%
[39]	United Kingdom	Any	94–'96	460	6.1%	9682*	1.3%
[40]	Brazil	Any	95–'97	142	13.4%	151	0.0%
[41]	Brazil	Any	96–'00	16	6.3%	76	6.6%
[42]	Brazil	Any	00–'02	72	2.8%	292	6.2%
[43]	Peru	All	'99–'00	81	43.2%	965*	3.9%
[44]	Haiti	Primary	'00–'02	115	9.6%	166	3.0%
		Acquired		16	0.0%	33	30.3%
[45]	New York City	Primary	'91	82	19.5%	145*	2.1%
[46]	New York City	Primary	'94	45	16.0%	104*	1.0%
[47]	New Jersey	Any	'91–'95	556	4.9%	413	1.2%
[48]	United States	Any	'92–'94				
	New York City	252	19.4%	69	5.8%		
	non-NYC			179	2.8%	473	1.4%
[49]	United States	Any	93–'96				
	US born		5375	6.4%	3929	1.4%	
	foreign born			957	4.7%	1867	3.0%
[50]	United States	Any	93–'94	4029	4.8%	21679**	0.6%
[51]	Texas	Any	'87–'96	2221	1.1%	15204**	1.4%

* includes patients with unknown HIV status.

** all patients have unknown HIV status.

For each eligible study, MDR-TB prevalence ratios and corresponding 95% confidence intervals were calculated from the reported data. Sample size, use of appropriate statistical measures to assess the measure of association between MDR-TB and HIV infection, assessment of potential confounders, and appraisal of external validity of study results were noted as quality indicators. Potential confounders included baseline demographic characteristics such as socioeconomic status, imprisonment and intravenous drug use.

The association between primary and acquired MDR-TB and HIV were assessed separately when possible. The standard chi-square test of homogeneity and associated p-value were calculated, and pooled prevalence ratios were generated, if appropriate, using the metan command in the STATA statistical software system [16]. Prevalence ratios were analyzed by region, study-period and date of publication.

## Results

**Figure 2:**
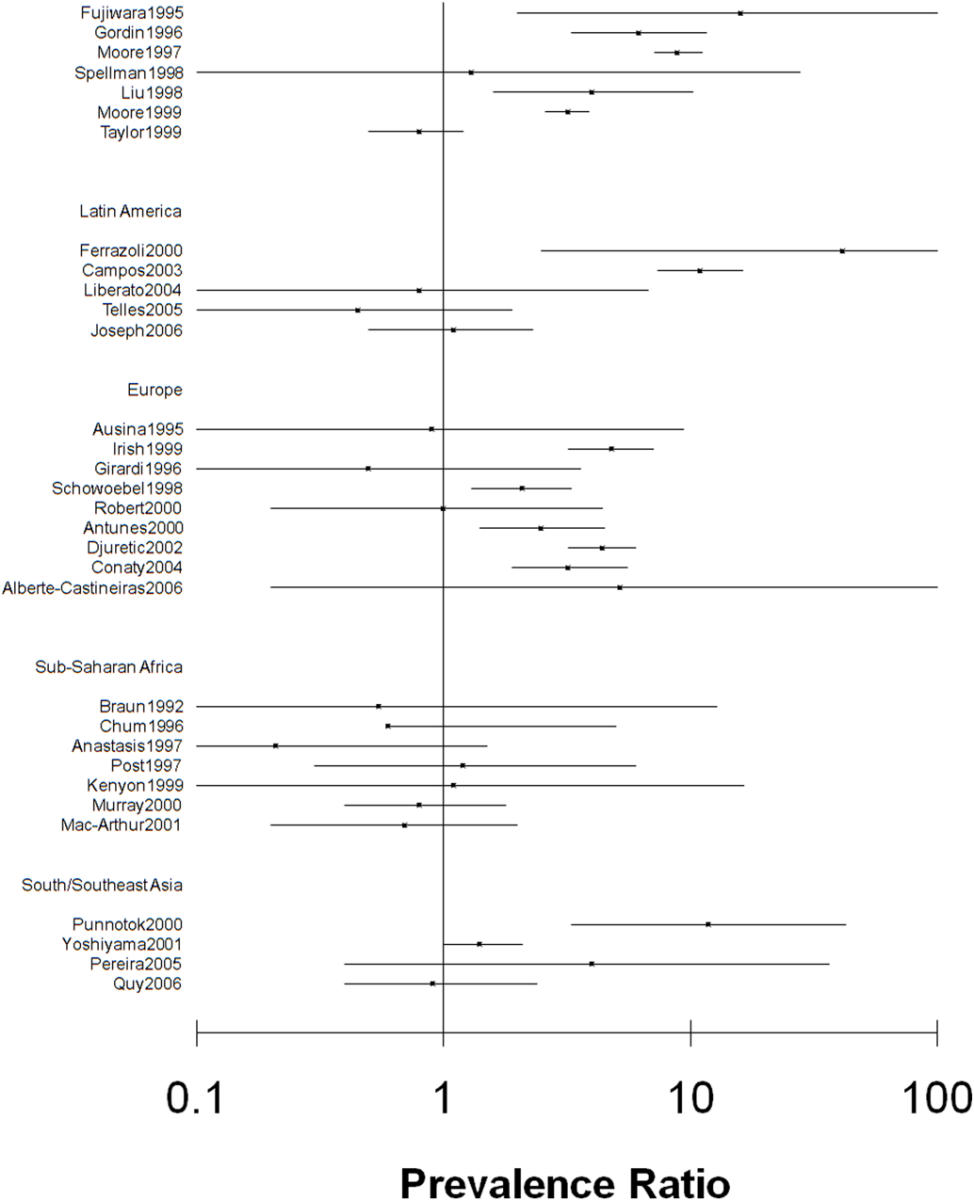
Forest plot of MDR-TB prevalence ratios by HIV status and corresponding 95% confidence intervals by geographical region^*^. ^*^Clark O; Djulbegovic B. Forest plots in excel software (Data sheet). 2001. Available at www.evidencias.com.

From PubMed, 738 articles concerned human subjects studies (Figure 1). ISI/Web of Science yielded 593 studies while the other databases yielded 30 additional studies. Eligibility criteria were met by 27 articles. One potentially eligible study published in *Problemy tuberkuleza i bolezneǐ legkikh* [17] was excluded because access could not be gained to the full article or data. References of the 26 articles and two review articles [18], [19] were hand-searched, yielding 6 additional eligible studies. The resulting 32 studies, which had study populations varying from 46 to 19,646, were carried out between 1988 and 2006, and represented 17 countries in 5 regions: South/Southeast Asia, Sub-Saharan Africa, Western Europe, Latin America, and North America (Table 1). Crude prevalence ratios and corresponding 95% confidence intervals are presented by region in Figure 2.

### South/Southeast Asia

In Bangkok, Thailand, an increased rate (RR 11.9, 95% CI 4.3, 33) of MDR-TB was found among 192 HIV co-infected patients, compared to 685 HIV-negative patients [20]. Groups were comparable for TB risk factors but only a small number of eligible patients were tested for HIV infection and TB drug susceptibility, which could have introduced selection bias.

A study in Northern Thailand found an association between HIV infection and primary MDR-TB (OR 2.0, 95% CI: 1.1, 3.5), but not acquired MDR-TB (OR 1.40, 95% CI: 0.68, 2.91) [21]. There was no baseline comparison of risk factors among the 426 HIV-positive and 559 HIV-negative patients.

A small (n = 70) Indian study observed similar rates of MDR-TB in HIV co-infected (10%) and HIV-negative patients (2.5%) [22].

A study from Vietnam showed no difference in primary MDR-TB prevalence rate among 40 HIV co-infected (7.5%) and 1393 HIV-negative patients (3.7%), even after adjustment for age, sex and treatment history. The rate of acquired MDR-TB was lower in 11 HIV co-infected (9.1%) than in 390 HIV-negative patients (25.9%) [23].

### Sub-Saharan Africa

Braun et al. undertook one of the first studies of antituberculosis drug resistance and HIV in sub-Saharan Africa [24]. In this small (n = 46) study from Cote d'Ivoire, MDR-TB rates among HIV co-infected (0%) and HIV negative (3.4%) patients were similar.

A large survey in Tanzania studied one-sixth of incident TB cases from each district over a three year period [25]. Among 1091 new TB cases, similar MDR-TB prevalence rates were found by HIV status (0.36% for co-infected and 0.37% for HIV-negative). Rates of acquired MDR-TB were 0% among 21 HIV co-infected and 3.8% among 52 HIV-negative patients. While no statistical testing was presented, the authors did provide limited baseline characteristics by HIV status.

Three studies in South Africa also found no association between HIV infection and MDR-TB. In a retrospective study in Durban, 2.4% of 42 HIV co-infected and 11.5% of 253 HIV-negative patients had MDR-TB [26]. A prospective study of hospitalized TB patients in Cape Town found a MDR-TB prevalence of 3.2% in 93 HIV co-infected patients, compared to 2.6% in 115 HIV-negative patients [27]. In goldminers, the MDR-TB rate was 5.3% among 207 HIV co-infected and 6.5% among 215 HIV-negative miners [28]. Only limited baseline characteristics and no statistical tests results were presented by these three studies.

A national survey using random sampling in Botswana also found similar MDR-TB prevalence rates among 107 HIV co-infected (0.9%) and 119 HIV-negative patients (0.8%) [29]. No information was provided on baseline characteristics by HIV infection status.

A study in Mozambique of 179 HIV co-infected and 530 HIV-negative TB patients, found an odds ratio of 0.7 (95% CI: 0.2, 2.2) for the association between MDR-TB and HIV infection. Baseline characteristics showed differences by HIV status in level of education, history of sexually transmitted diseases, and history of TB treatment [30].

### Europe

A study of hospitalized TB patients in Rome, Italy found that 2.9% of 34 HIV co-infected patients and 5.9% of 373 patients without documented HIV status had MDR-TB (OR 0.5, 95% CI: 0.1–3.2) [31]. Patient characteristics were not compared by HIV status.

A Portuguese study found a higher MDR-TB rate among 29 HIV co-infected patients (44.8%) compared to 17.7% among 113 HIV-negative patients [32].

Two studies from Spain addressed the issue of MDR-TB and HIV infection. In Barcelona, the primary MDR-TB rate was similar among 184 HIV co-infected (0.5%) and 317 HIV-negative patients (0.6%). Statistical testing was not provided and potential confounders were not assessed [33]. In the Spanish Castilla-Leon region, equally low rates of MDR-TB were observed among HIV co-infected (0%) and HIV-negative patients (0.1%) (OR 0.19, 95% CI: 0, 4.8) [34]. Comparison of baseline characteristics by HIV status was not addressed.

A large survey (n = 13,344) conducted in the early 1990's in France, representing 80% of French public hospital beds, observed an association between primary MDR-TB and HIV (OR of 3.3; 95% CI: 1.5, 7.3), but not for acquired MDR-TB (OR 1.0; 95% CI 0.5, 2.0) when adjusted for sex, age, and region of origin [35]. A smaller (n = 2998) French survey found no statistically significant association between primary or acquired MDR-TB and HIV infection [36].

Three surveillance studies with some overlapping data from the United Kingdom have been published. A survey of 910 HIV co-infected patients and 24,307 HIV-negative patients found an odds ratio of 4.6 (95% CI 3.3, 6.2) for MDR-TB by HIV status [37]. Another study found an association between HIV infection and primary MDR-TB (OR 3.6, 95% CI 1.8, 7.0), but no association between acquired MDR-TB and HIV infection (OR 2.2; 95% CI 0.7, 6.9) [38]. A third study suggested higher MDR-TB rates among HIV-infected patients (6.1% vs. 1.3%) [39]. None of these three studies presented patient characteristics by HIV status.

### Latin America

Three Brazilian studies assessed MDR-TB rates by HIV status. One study suggested a higher rate (13.4% vs. 0%) of MDR-TB (13.4%) among 142 HIV co-infected compared to 151 HIV-negative patients [40]. Gentoype patterns suggested that most MDR-TB cases were recently transmitted infections. Baseline characteristics were not addressed by HIV status. A small (n = 92) study found no relationship between MDR-TB rates by HIV status (6.3% among HIV co-infected and 6.6% among HIV-negative patients) [41]. The two groups differed regarding proportion male, injection drug use, and male homosexual relations. A subsequent cross sectional study also found no relationship between MDR-TB and HIV infection, with 2.8% of 72 co-infected patients and 6.2% of 292 HIV-negative patients having MDR-TB [42].

A study in Peru found an association between HIV infection and MDR-TB, with 43.2% of 81 HIV co-infected and 3.9% of 965 HIV-negative patients having a diagnosis of MDR-TB [43]. Important differences between groups included socio-economic status, TB treatment history, TB exposure, and use of medical services in the year preceding active TB. Important to note is that HIV co-infected patients were recruited from hospitals, whereas HIV negative controls were recruited from ambulatory clinics.

In Haiti, a study found an increased risk for primary MDR-TB among the 115 patients with HIV co-infection (RR 3.2, 95%CI 1.1, 8.9), but no increased risk for acquired MDR-TB. Prevalence of acquired MDR-TB was actually higher among HIV-negative patients [44]. Baseline characteristics were not compared by HIV status.

### United States

In 1993, HIV infection was found to be highly correlated with MDR-TB in New York City, with 19.5% of HIV co-infected patients diagnosed with MDR-TB compared to 2.1% of those without documented acquired immune deficiency syndrome (P<0.001) [45]. There was no assessment of confounding. A smaller survey from New York in 1994 also showed an association between MDR-TB and HIV infection (OR 61.7) [46]. Similarly, in nearby New Jersey, HIV co-infected patients were at higher risk of MDR-TB (OR 3.6, 95% CI: 1.5, 8.8) [47]. Factors such as previous TB, homelessness, and injection drug use were examined, but not stratified by HIV status.

Another study observed an increased rate of MDR-TB among HIV co-infected patients in New York (19.4% among 252 HIV co-infected vs. 5.8% among 69 HIV-negative), but not among patients residing outside New York City (2.8% among 179 HIV co-infected vs. 1.4% among 473 HIV-negative) [48]. There was no comparison of baseline characteristics by HIV status. Analysis of nationwide TB surveillance data, including a subset of the patients reported in Gordin et al, showed differences in MDR-TB rates by HIV status for both US-born (6.4% among 5375 HIV co-infected vs. 1.4% among 3929 HIV-negative) and foreign-born patients (4.7% among 957 HIV co-infected vs. 3.0% among 1867 HIV-negative) [49]. A multivariate model that included age, TB history, country of birth, residence in New York City, and race/ethnicity, found HIV infection to be a risk factor for MDR-TB, with rates of 4.8% among 4029 HIV co-infected and 0.6% of 21679 with unknown HIV status [50]. In some areas of the United States with low levels of MDR-TB, differences in MDR-TB by HIV status were not found. A study in Texas found a relative risk for MDR-TB by HIV status of 0.78 [95% CI: 0.50, 1.21] [51].

### Summary Prevalence Ratio

**Figure 3:**
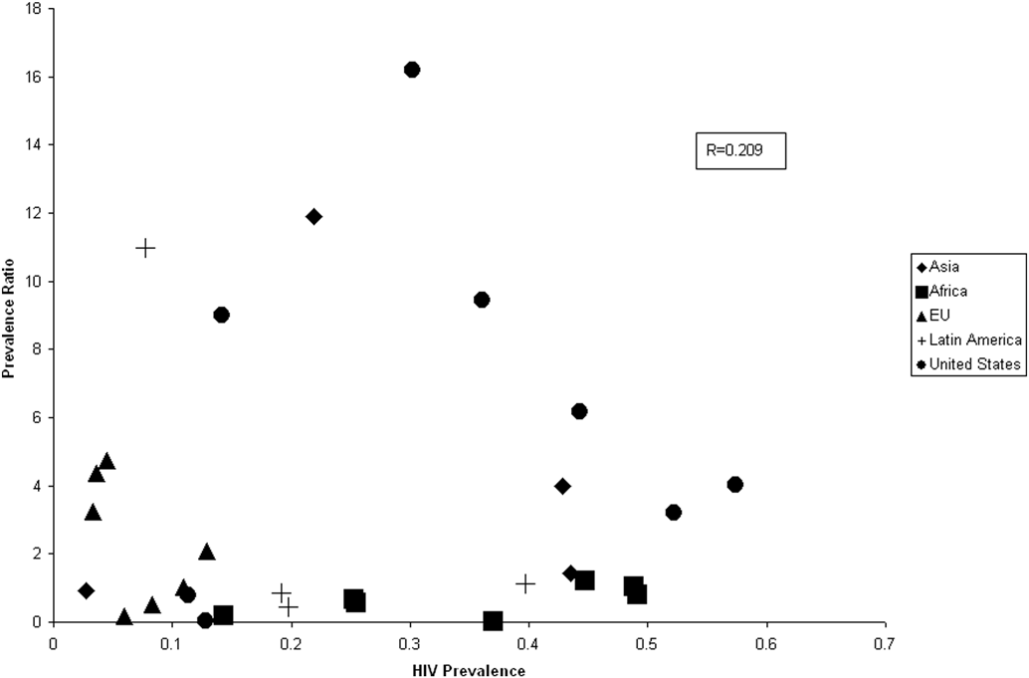
MDR-TB Prevalence ratio by HIV prevalence among study participants and by region^*^. ^*^One outlier from the Latin American region (HIV Prevalence: 0.20, Prevalence Ratio: 45) is not presented.

The MDR-TB prevalence ratio in individual studies ranged from 0.21 to 41.45 and varied widely between studies, between regions and within regions (Figure 2). The HIV prevalence also varied widely between studies (range 3 to 57%), between regions (mean 12% to 33%) and within regions. There was no overall correlation between HIV prevalence and MDR-TB prevalence ratio (r = 0.209) (Figure 3).

**Figure 4:**
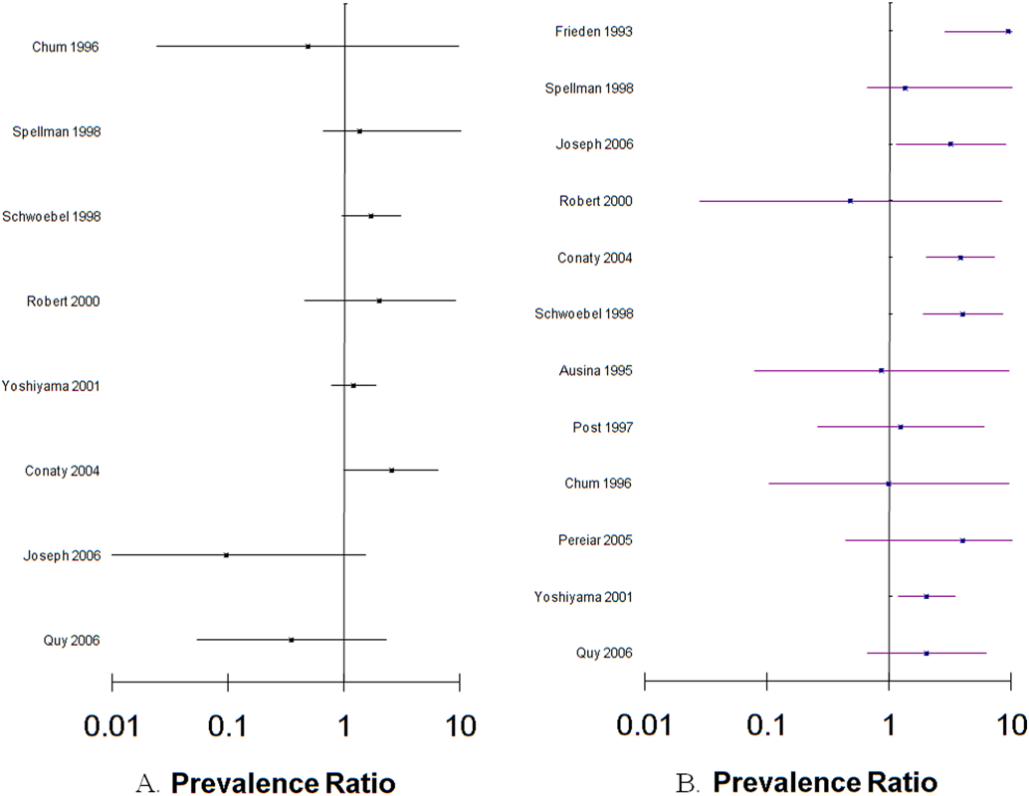
Forest plots of acquired (A) and primary (B) MDR-TB prevalence ratios by HIV status and corresponding 95% confidence intervals^*^. ^*^Clark O; Djulbegovic B. Forest plots in excel software (Data sheet). 2001. Available at www.evidencias.com.

The heterogeneity across studies did not allow for the calculation of one summary prevalence ratio for the relationship between MDR-TB and HIV infection, despite accounting for region and study period. The pooled prevalence ratio for acquired MDR-TB and HIV (8 studies) was 1.17 (95% CI 0.86, 1.6, p-value for heterogeneity = 0.188), the pooled prevalence ratio for primary MDR-TB and HIV (12 studies) was 2.72 (95% CI 2.03, 3.66, p-value for heterogeneity = 0.356) (Figure 4).

## Discussion

While most studies in North America showed an association between HIV infection and MDR-TB, not a single study from Africa demonstrated such association, and results from other regions were conflicting. Individual studies varied widely in study design and sample size, and results were rarely adjusted for potential confounding. These limitations and the observed heterogeneity precluded a general conclusion regarding the overall association between HIV infection and MDR-TB. When stratified by type of MDR-TB, the analysis suggests that primary, but not acquired, MDR-TB is associated with HIV infection.

Several biological mechanisms linking drug resistant TB to HIV infection have been suggested [52]. Drug malabsorption in HIV-infected patients, especially rifampin and ethambutol, can lead to drug resistance and has been shown to lead to treatment failure [53]. Drug resistant strains may be less virulent and preferentially lead to disease progression in immune compromised patients, as opposed to immune-competent individuals. Data supporting this hypothesis has not yet been observed in humans.

However, the association between HIV infection and MDR-TB could be confounded by other factors. First, an observed association could be confounded by time window. HIV-negative patients are likely to reactivate a latent infection from decades ago, whereas HIV infected patients, in whom disease progresses rapidly, are likely to reactivate an infection acquired more recently following community or institutional transmission. With increasing prevalence of drug resistance globally [54], a higher percentage of recent infections are likely to be multi-drug resistant, resulting in higher rates of MDR-TB in HIV-positive individuals. The significant associations between HIV infection and primary MDR-TB, but not between acquired MDR-TB and HIV infection, support this [21], [35], [38], [44]. Similarly, the observed association between XDR-TB and HIV infection may be a result of the recent evolution of XDR-TB [13].

Second, the association between HIV infection and MDR-TB may be confounded by shared risk factors such as injection drug use, imprisonment, socioeconomic status, alcohol use and hospitalization. Intravenous drug use is a risk factor for HIV infection and non-adherence to treatment, the latter promoting development of drug resistance [55]. HIV-infected patients and MDR-TB patients are more likely to be hospitalized compared to those who are HIV negative or suffer from drug sensitive TB. HIV-infected patients may thus be more likely to be exposed to patients with drug resistant isolates, and thus be infected or re-infected with a resistant isolate. The associations between MDR-TB and HIV infection observed in many North American studies, which included in part patients involved in institutional outbreaks in New York City, support this possibility.

Several limitations restrict the interpretation of our findings. Publication bias, an important concern in meta-analysis of randomized control trials, may have been present but was most likely not substantial as many studies were not designed to assess the relationship between MDR-TB and HIV infection, and several studied included in this review observed no relationship between MDR-TB and HIV infection. The quality of many studies was poor due to small sample size and lack of adjustment for possible confounders in all but 7 studies. As the primary aim of many studies was not to assess the association between HIV infection and MDR-TB, studies could have suffered from misclassification bias due to inclusion of patients with unknown HIV status, and participation bias when HIV infected individuals were more likely to be tested for drug resistance [18]. Level of immunosuppression (CD4 count) was not presented, and most studies were performed prior to the introduction of highly active antiretroviral therapy. Data from MDR-TB hot-spots, such as Russia, Eastern Europe, and China, were not available. One potentially eligible study from the Samara region in Russia was excluded due lack of access to the data. Inclusion of this article would most likely not have changed our conclusion as the abstract states that “HIV infection is unassociated with resistance”. Finally, studies not published in peer reviewed journals were excluded. Two reports deserve special attention. A large South African survey did not find a difference in MDR-TB rates by HIV status (p = 0.575) [56]. In the 2008 report on antituberculosis drug resistance in the world [9], only 7 of 81 countries reported data in drug resistance stratified by HIV status, and only Latvia and one oblast in Ukraine (Donetsk Oblast) reported large enough numbers to examine the association between HIV infection and MDR-TB. MDR-TB was associated with HIV infection in both Latvia (OR 2.1; 95% CI 1.4, 3.0) and Ukraine (OR 1.5, 95% CI 1.1, 2.0), but HIV negative and HIV unknown were not distinguished in Latvia. In Donetsk Oblast, HIV infection was found to be an independent predictor for MDR-TB, in addition to history of previous TB treatment and history of imprisonment [57].

We opted not to present a pooled prevalence ratio for the overall association between HIV infection and MDR-TB because of the high degree of heterogeneity among studies. To provide the least biased estimate of the association between HIV infection and MDR-TB, future prospective studies will need to be sufficiently powered and will need to measure HIV status, drug resistance, history of TB treatment, and potential confounders in all eligible patients with TB.

While HIV infection and tuberculosis are intimately linked, there is currently no evidence supporting an association between MDR-TB and HIV outside of institutional outbreaks. Nevertheless, the devastating consequences of HIV and MDR-TB co-infection and the geographic overlap of these two epidemics demand urgent attention [18], [19]. The high case fatality rates of MDR and XDR-TB in HIV co-infected patients could have devastating and demoralizing effects on health care workers and communities. Concomitant MDR-TB and antiretroviral treatment requires adherence to 6 to 10 daily medications for more than one year, and is characterized by high levels of toxicity and drug-drug interactions, leading to increased complexity of patient management. Limited infection control, lack of access to MDR-TB diagnostics, and poor MDR-TB treatment capacity in resource poor settings may lead to hospital outbreaks, similar to what had been observed in New York almost two decades ago [45], and in South Africa more recently [13].

Intervention, as outlined by the WHO Stop TB Strategy, is needed to avert a global catastrophe of a dual HIV and MDR-TB epidemic. Basic TB control needs to be strengthened to achieve high cure rates and prevent ongoing generation of MDR-TB. Laboratory capacity for prompt diagnosis of drug resistance, preferably using rapid diagnostic techniques such as the GenoType MTBDR*plus* [58], needs to be built. Capacity for concomitant MDR-TB and antiretroviral treatment needs to be scaled up, and collaborations between HIV and TB control programs need to be strengthened. Infection control measures need to become a key element of global TB control.

In addition, gaining a better understanding of how HIV infection impacts the epidemiology of drug resistant TB will be critical. The public health and medical community should not have to rely on small, isolated surveys for two of the largest threats to TB control. Surveillance of MDR-TB and XDR-TB among HIV positive and negative patients should be an essential part of global efforts to combat TB and HIV. Universal access to diagnosis and treatment of HIV and drug resistant TB should be the ultimate goal. To achieve this, a united front will be needed to help avert “the perfect storm” [19] of a massive MDR-TB/HIV co-epidemic, which may turn out to be greater than the sum of its components.
